# Analysis of clinical factors impacting recurrence in myxofibrosarcoma

**DOI:** 10.1038/s41598-024-53606-y

**Published:** 2024-02-16

**Authors:** Wenlin Chen, Ming Ye, Ye Sun, Yongzhong Wei, Yumin Huang

**Affiliations:** 1 Department of Orthopedics, The First Affiliated Hospital of Nanjing Medical University, Nanjing, 210029 China; 2 Nanjing Medical University, Nanjing, 210029 China

**Keywords:** Cancer, Diseases, Oncology, Risk factors

## Abstract

Myxofibrosarcoma (MFS) is a malignant fibroblastic/myofibroblastic neoplasm with a prominent myxoid area. It has the clinical features of frequent local recurrence (LR) and occasional distant metastasis. Robust epidemiological data on MFS in China are lacking. The aim of this retrospective analysis was to determine the natural history of MFS, identify prognostic factors for recurrence and describe the real-life outcomes of MFS. We reviewed 52 patients with primary MFS from the First Affiliated Hospital of Nanjing Medical University diagnosed between 2016 and 2020. All tumors were subjected to retrospective univariate analysis for prognostic factors of the disease, including tumor size, grade, location and sex; patient age; planned operation; surgical margin; and laboratory results. The significant factors identified by univariate analysis were subsequently analyzed via multivariate analysis. Overall survival (OS), post-treatment LR and metastatic-free survival were assessed as outcomes. The median age was 61 years (range, 13–93). Fourteen (26.92%) patients exhibited low grade disease, and 38 (73.08%) exhibited high grade disease. Among the 29 males, and 23 females, 15 (28.85%) had tumors in the trunk, 37 (71.15%) had tumors in the extremities, 26 had undergone planned surgery, and 26 had unexpected unplanned operation. The margin was negative in 39 (75%) patients and positive in 13 patients (25%). The serum creatine kinase (CK) concentration was high level in 33 (63.46%) patients and low level in 19 (36.54%) patients. The serum lactate dehydrogenase (LDH) levels were low in 23 (44.23%) patients and high in 29 (55.77%) patients. LR was observed in 25 patients (48.08%), and 4 patients developed metastasis. A worse LR rate was found for patients with a low CK level (84.21%) than for those with a high CK level (27.27%) at 5 years (*p* < 0.05). The LR rate of patients who underwent planned surgery was lower than that of patients who underwent unplanned surgery (*p* < 0.05). There were significantly more patients with positive margins than patients with negative margins (92.30%, and 33.33%, respectively; *p* < 0.05). Moreover, superficial tumors were also associated with greater recurrence rate (2/20 [10%]) than deep tumors, (23/32 [71.86%]) [*p* < 0.05]. The probability of LR in patients with MFS was significantly greater in association with unplanned operations, positive margins, low serum CK levels or superficial tumor depth. These data could help identify high-risk patients; thus, more careful follow-up should be performed for higher-risk patients. Diagnosis and treatment at qualified regular medical centers can reduce the local recurrence rate of MFS.

## Introduction

Myxofibrosarcoma (MFS) constitutes a specific subtype of mesenchymal malignancy, and represents approximately 5% of all soft tissue sarcomas (STSs)^1^. This malignancy predominantly occurs in the extremities and is typically characterized by a gradually enlarging, asymptomatic mass. Historically, MFS was encompassed within the category of malignant fibrous histiocytoma. Nonetheless, in 2002, the World Health Organization (WHO) acknowledged MFS as an independent diagnostic entity. This reclassification was considered imperative owing to the distinctive attributes that demarcate the myxoid variant of malignant fibrous histiocytoma as a separate entity^2^.

Owing to the rarity of MFS and its comparatively recent reclassification by the WHO, there is a pronounced deficiency in exhaustive epidemiological data concerning the condition. At present, a restricted number of studies have been performed, delineating a five-year overall survival (OS) ranging between 61 and 77%^3–5^. Within the clinical context, MFS is typified by an infiltrative growth pattern, extending into contiguous connective tissues, complicating the assessment of postsurgical tumor dimensions^6^. This particular growth pattern engenders an elevated inclination for local recurrence (LR) in MFS patients compared to that in other STS patients, with documented rates varying between 14 and 61%, in contrast to approximately 10% in alternative STS classifications^7,8^. As a result, amputation is a more prevalent therapeutic approach for MFS than for other STSs^1^. Moreover, it is noteworthy that positive margins are presently acknowledged as detrimental factors contributing to recurrence. Nevertheless, a contentious issue arises concerning the impact of various other clinical factors on the recurrence of MFS, primarily due to the limited availability of cases for comprehensive analysis.

The incidence of metastatic disease in patients with MFS has been documented in the literature, with reported rates ranging between 15 and 38%. Factors such as histological grade and tumor size have been proposed as potential determinants of metastatic risk. Unfortunately, in the event of metastasis, therapeutic options specific for MFS are unavailable, rendering the prognosis dire.

Our study was designed to augment the limited epidemiological data pertaining to MFS. Concentrating on the period between 2016 and 2020, we aimed to meticulously document patient demographics, tumor characteristics, laboratory results, and clinical outcomes from MFS patients diagnosed at the First Affiliated Hospital of Nanjing Medical University. Notably, our primary focus was on a meticulous analysis of the pivotal factors that influence the recurrence patterns in these patients.

## Materials and methods

### Study design and setting

This study was an institutional review board-approved single-institution retrospective chart review of the electronic medical records (EMRs) of consecutive STS patients. Prior to the data collection, we obtained the approval of The Ethics Committee of The First Affiliated Hospital of Nanjing Medical University (ethical review number:2023-SR-941). And the informed consent of all participating patients was obtained. All experiments were performed in accordance with relevant guidelines and regulations.

### Participants

A review of patient records from our institutional EMR allowed us to determine the underlying patient and tumor characteristics and eligibility for the study. Patients with a diagnosis of MFS who presented for initial tumor resection or tumor bed re-excision between 2016 and 2020 and who had at least one postsurgical follow-up were eligible for participation. We included trunk and extremity tumors of all sizes and depths.

**Figure 1:**
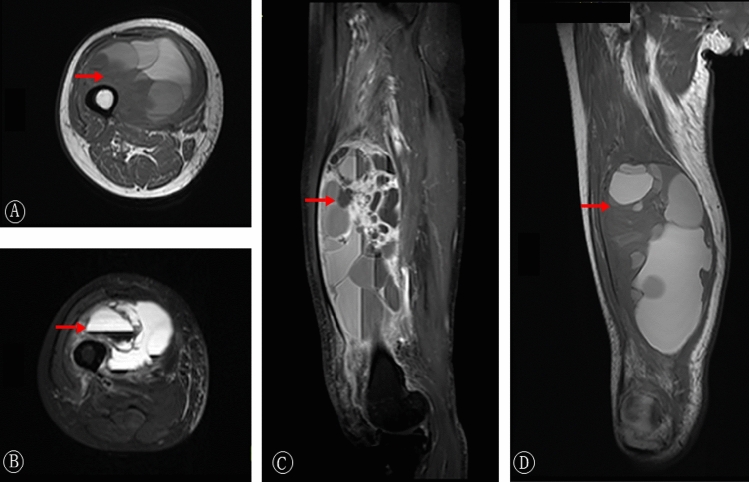
A 58-year-old woman with 16 cm myxofibrosarcoma of the right thigh. The round-like soft tissue occupied the right anteromedial femur, and the signal was uneven (**A**–**D**). Multiple round-like segment T1 signals (**A**, **D**) or long T2 signals (**B**, **C**) were seen inside, and the edge separation was enhanced after enhancement (**B**).

**Figure 2:**
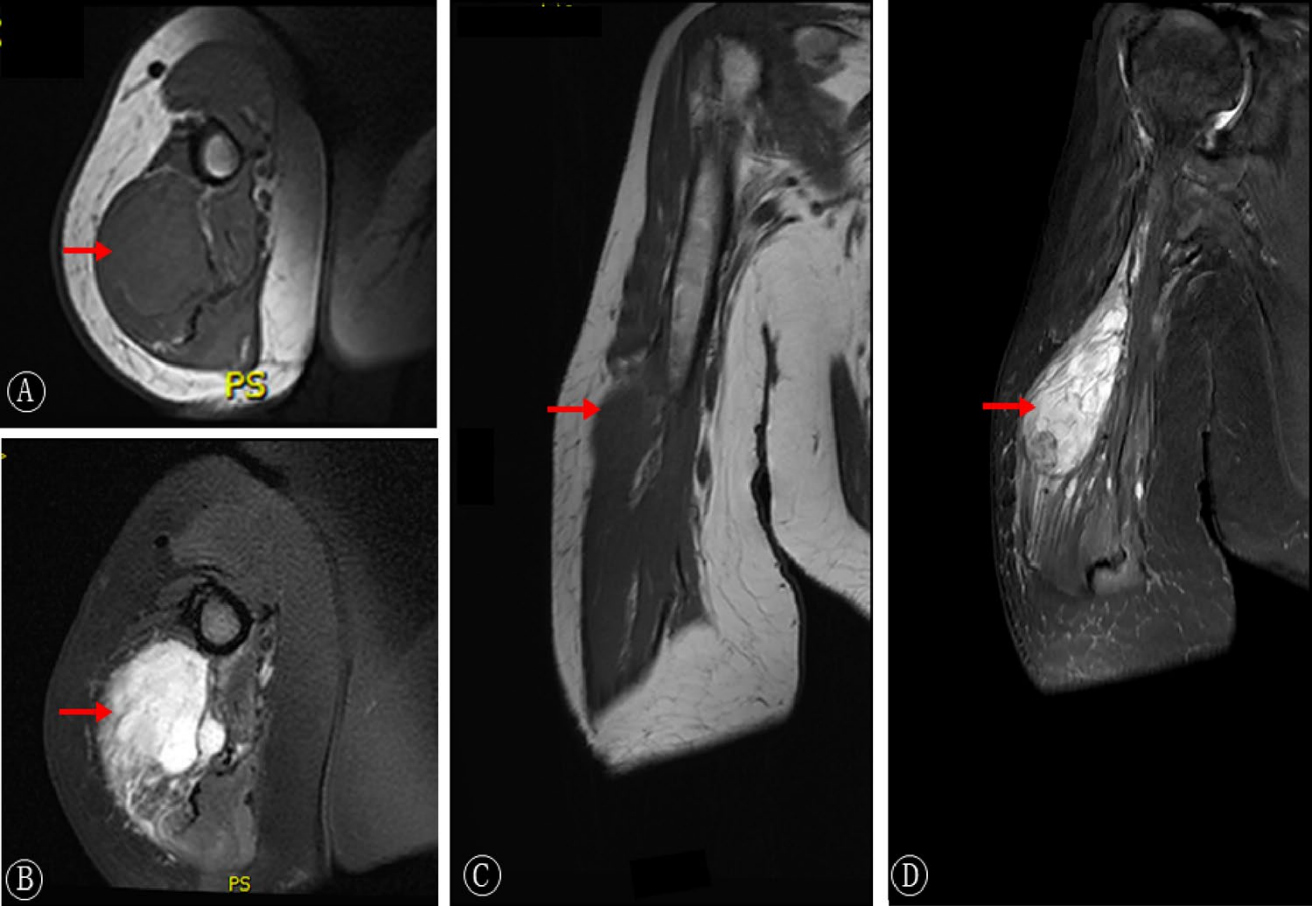
An 84-year-old woman with 8 cm myxofibrosarcoma of the right upper arm. Irregular long T1 and long T2 signal masses were seen in the right triceps muscle with multiple septum and uneven enhancement.

In accordance with the patient selection process, patient and treatment data were collected from the hospital EMR. A multidisciplinary tumor board with fellowship-trained specialists from various fields reviewed the tumor data of each patient. Pathological was performed by two pathologists at our hospital, when there was a disagreement, a third pathologist was consulted, and a conclusion was finally reached. Our pathologists employed the histologic criteria outlined in the 2013 WHO Classification of Soft Tissue Tumors to diagnose MFS. According to this classification, MFS is a malignant mesenchymal tumor characterized by a multinodular proliferation of spindle-shaped to polygonal tumor cells displaying varying degrees of nuclear atypia. These features are accompanied by a myxoid background, delicate slit-like blood vessels, and structures referred to as "pseudolipoblasts." To differentiate MFS from undifferentiated pleomorphic sarcoma (UPS), we followed the approach proposed by Yoshimoto^9^, defining MFS as having a mucinous component comprising ≥ 10% of the tumor's maximum cross-sectional area. Additionally, we conducted immunohistochemical staining for MDM2 and/or MDM2 gene fluorescence in situ hybridization for all tumors to exclude the possibility of myxoid liposarcoma^10–13^. Patients exhibiting immunohistochemical positivity for MDM2 and/or MDM2 gene amplification were subsequently excluded from the analysis. The radiologic evaluation consisted of contrast-enhanced magnetic resonance imaging (MRI) (Figs. 1 and 2), including gadolinium-enhanced sequences for surgical planning, and at a minimum, computed tomography of the chest and a full-body functional nuclear scan (FDG-PET) to assess regarding the presence of metastatic disease.

After the patients were transitioned from diagnosis to treatment, all patients, irrespective of their presentation status, underwent full staging and interventions according to the standard treatment protocol of our sarcoma center. This protocol consists of neoadjuvant radiotherapy followed by wide resection. Patients were stratified into two groups based on their presentation, and unplanned resection was defined specifically for the purpose of this study.

Patient data, including patient demographics, recurrence, survival, laboratory results, and tumor characteristics, were acquired from the EMR. Radiation and chemical treatment, timing, and dose were also recorded. Before the operation, we planned for the edge of all MRI enhancement areas to be 2 cm, and the final surgical margins were determined by an institutional pathologist. The postoperative follow-up protocol adhered to institutional guidelines in line with the European Society for Medical Oncology (ESMO) Clinical Practice Guidelines^14^. The schedule involved assessments every 3–4 months during the initial 2–3 years, followed by semiannual evaluations up to the fifth year, and subsequently, annual assessments. Each examination included local enhanced magnetic resonance imaging, while chest X-rays or CT scans were taken at longer intervals during the initial 3–5 years and then annually thereafter.

Finally, our primary endpoint was LR, which was documented in the EMR or through outpatient follow-up. MRI and ultrasound were used routinely for local surveillance, and every patient suspected of having LR was subjected to histological confirmation. Participants who did not experience local recurrence were censored at the time of the latest clinical evaluation. The time to local recurrence was defined as the time from surgery to the time of recurrence, and participants were considered to have a competing risk of mortality for the time-based analysis.

### Statistical analysis

Patient demographics comparing local recurrences in the entire cohort and local recurrences in patients were modeled utilizing the Chi-square or Fisher’s exact test for categorical variables and the Wilcoxon sum rank test for continuous variables, as appropriate. The log-rank test was used to compare differences in survival between different groups. Patients were censored at the time of last follow-up if they had not experienced an LR. Local recurrence was modeled using the Cox proportional hazard ratio to estimate the independent recurrence of the predicted variables and was adjusted for age, sex, grade, size, site, prior surgery, laboratory results, and margins, with considerations regarding the competing risk of mortality. The statistical analyses were performed using IBM SPSS statistics, version 25. A *p*-value of < 0.05 was considered indicative of statistical significance.

## Results

### Patient and clinical characteristics

We identified 52 patients (19 patients were excluded—15 due to loss to follow-up and an additional 4 due to conditions such as myocardial disease and dermatomyositis.) who met the inclusion criteria with a median follow-up of 52 months (range 36–88 months) and a median age of 61 years (range, 13–93). 55.77% of the patients were male (29/52). There was no record of previous cancer in any of the patients' medical histories. Most of the tumors were high grade (73.08%,38/52), and 14 were low grade (26.92%). The extremities were the most common primary location (71.15%, 37/52), followed by the trunk (28.85%, 15/52). The serum creatine kinase concentration was high level in 33(63.46%) patients and low level in 19(36.54%) patients. The serum lactate dehydrogenase (LDH) levels were low in 23(44.23%) patients and high in 29(55.77%) patients. For primary treatment, 26 patients underwent planned surgery, and 26 patients underwent unexpected surgery, resulting in no residual disease in 75% (39/52) of the patients. R1/R2 resections were reported in 25% (13/52) of the patients. Additional treatment with radiotherapy was given to 10 patients (19.23%), among them, 3 of the10 patients experienced recurrence. One patient received adjuvant radiotherapy and chemotherapy. LR occurred in 25 patients and 4 patients developed metastasis. See Table 1 for details.

**Table 1:** Clinicopathologic and treatment characteristics of 52 patients with Myxofibrosarcoma.

Variables	Overall, n (%)
Sex	
Male Female	29(55.77) 23(44.23)
Age	
> 60 years < 60 years	31(59.62) 21(40.38)
Location	
Trunk Extremity	15(28.85) 37(71.15)
Depth	
Superficial Deep	32(61.54) 20(38.46)
Size(cm)	
< 5 > 5	13(25.00) 39(75.00)
Histological grade	
I II-III	14(26.92) 38(73.08)
Residual disease	
R0 R1/R2	39(75.00) 13(25.00)
Surgery	
Planned surgery unplanned surgery	26(50.00) 26(50.00)
Local recurrence	
Recurrence No recurrence	25(48.08) 27(51.92)
Distant metastases	
Metastases No metastases	4(7.70) 48(92.30)
Radiotherapy	
NO Adjuvant	42(80.77) 10(19.23)
CK	
High Low	33(63.46) 19(36.54)

### Local recurrence rate

Univariate analyses revealed that low CK levels (*p* < 0.01), superficial tumor depth (*p* < 0.01), and R1 or R2 residual disease (*p* < 0.01), were correlated with a poorer LR rate (Table 2). However, sex, age, tumor size, histological grade and LDH level were not significantly different between the two groups.

**Table 2:** Univariate analysis of prognostic factors for local recurrence in 52 Myxofibrosarcoma patients.

Variables	Number of patients (%)	OR, HR (95%CI)	P-*value*
Sex			
Male Female	29(55.77) 23(44.23)	1.34(0.45–4.02) Ref	0.59
Age			
> 60 years < 60 years	31(59.62) 21(40.38)	Ref 0.99(0.96–1.03)	0.93
Location			
Trunk Extremity	15(28.85) 37(71.15)	4.52(1.20–16.97) Ref	0.02
Depth			
Superficial Deep	32(61.54) 20(38.46)	23.00(4.41–119.96) Ref	< 0.01
Size(cm)			
< 5 > 5	13(25.00) 39(75.00)	Ref 1.68(0.47–6.07)	0.43
Histological grade			
I II-III	14(26.92) 38(73.08)	Ref 2.00(0.56–7.09)	0.28
Residual disease			
R0 R1/R2	39(75.00) 13(25.00)	Ref 24.00(2.81–205.19)	< 0.01
Surgery			
Planned surgery Unplanned surgery	26(50.00) 26(50.00)	Ref 9.04(2.57–31.84)	< 0.01
Distant metastases			
Metastases No metastases	4(7.70) 48(92.30)	0.33(0.03–3.44) Ref	0.36
Radiotherapy			
NO Adjuvant	42(80.77) 10(19.23)	Ref 0.39(0.09–1.72)	0.39
CK			
High Low	33(63.46) 19(36.54)	Ref 14.22(3.33–60.73)	< 0.01
LDH			
High Low	29(55.77) 23(44.23)	Ref 0.54(0.18–1.64)	0.28

In addition, we investigated whether planned surgery at a dedicated sarcoma expertise center influenced the patient LR rate compared to unplanned surgery at a non-sarcoma specialized hospital. The LR rate significantly improved when the patients underwent planned surgery at a sarcoma expertise center rather than at a non-sarcoma center (*p* < 0.01).

**Figure 3:**
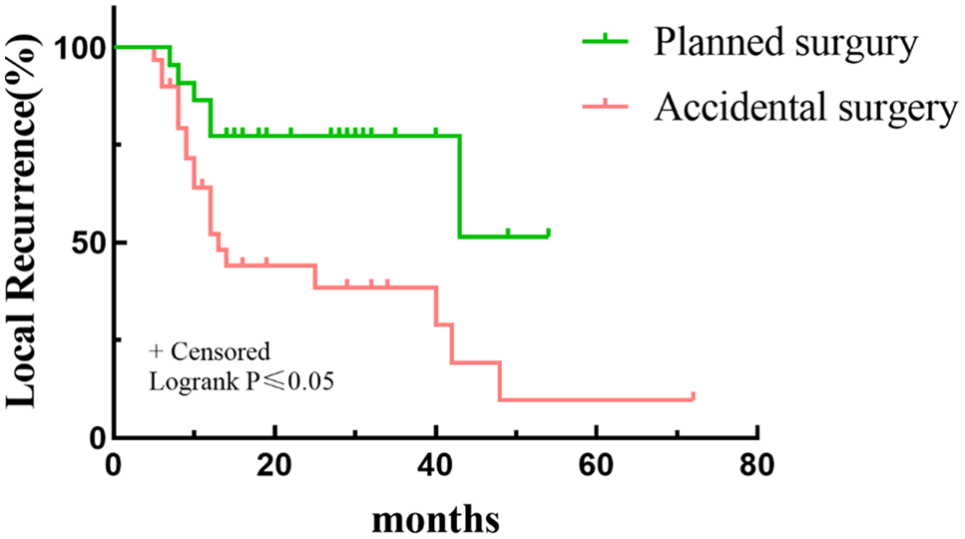
Kaplan–Meier Survival to local recurrent Plot. Comparison of recurrence rates of patients undergoing planned and unplanned surgery.

**Figure 4:**
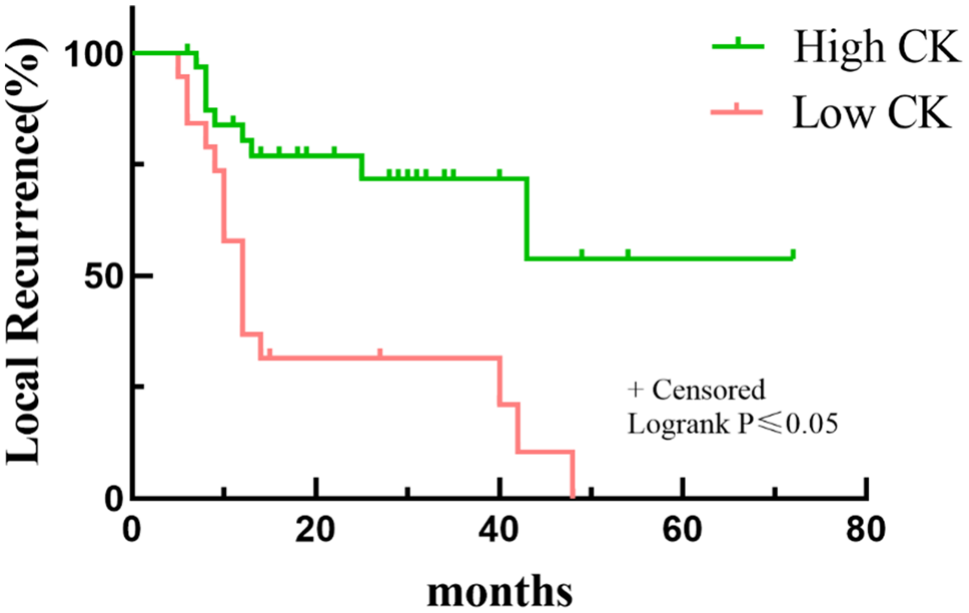
Kaplan–Meier Survival to local recurrent Plot. Comparison of recurrence rates of patients with high CK level or low CK level.

**Figure 5:**
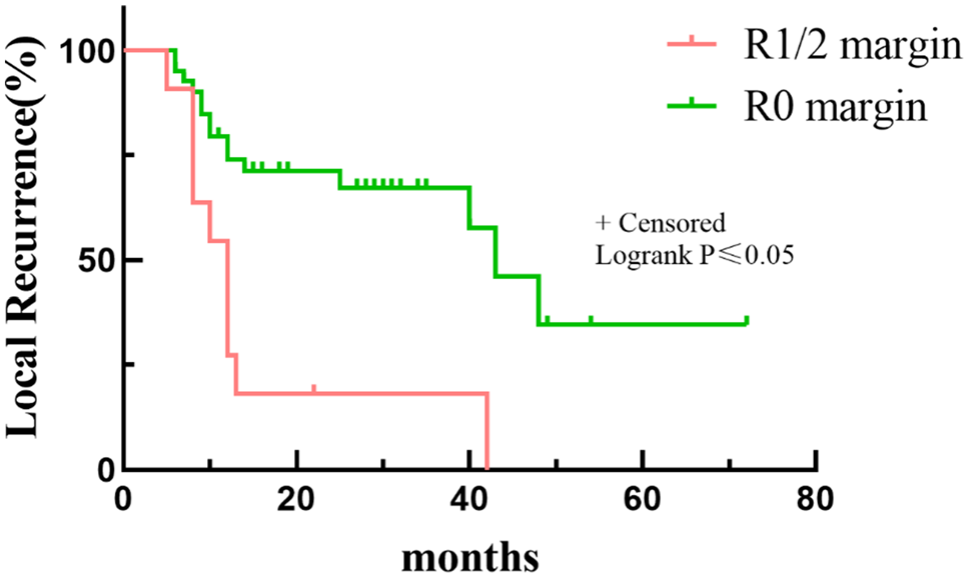
Kaplan–Meier Survival to local recurrent Plot. Comparison of recurrence rates of patients with margin of R0 or R1/2.

**Figure 6:**
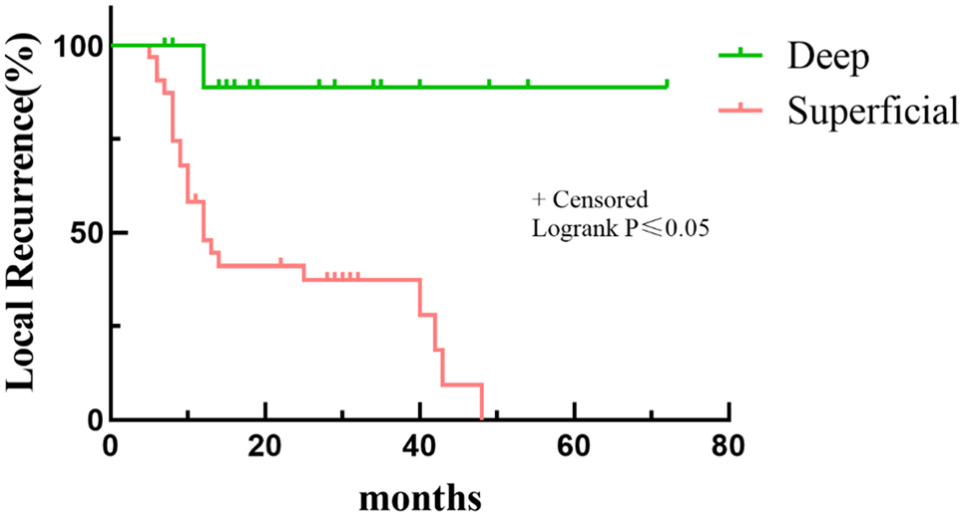
Kaplan–Meier Survival to local recurrent Plot. Comparison of recurrence rates of patients with tumor located in deep or superficial.

Multivariate analyses revealed that patients who underwent planned surgery had a better LR rate than patients who underwent unplanned surgery (*p* < 0.05) (Table 3 and Fig. 3). In addition, a worse LR rate was found for patients with a low CK level (84.21%) than for those with a high CK level (27.27%) at 5 years (*p* < 0.05, Fig. 4). Depth and residual disease were found to be independent prognostic factors, while having a tumor located in the superficial tissue and having an R1/R2 margin significantly increased the probability of tumor recurrence (*p* < 0.05 Figs. 5 and 6).

**Table 3:** Multivariate analysis of 52 Myxofibrosarcoma patients.

Variables	Number of patients (%)	OR	*P*-*value*
Location			
Trunk Extremity	15(28.85) 37(71.15)	0.1(0.004–2.51) Ref	0.16
Depth			
Superficial Deep	32(61.54) 20(38.46)	135.77(2.21–8352) Ref	0.02
Residual disease			
R0 R1/R2	41(78.85) 11(21.15)	Ref 24.39(1.02–581.55)	0.04
Surgery			
Planned surgery Unplanned surgery	22(42.31) 30(57.69)	Ref 44.78(1.81–1107.30)	0.02
CK			
High Low	33(63.46) 19(36.54)	Ref 99.23(1.01–9751.94)	0.04
LDH			
High Low	29(55.77) 23(44.23)	Ref 0.65(0.05–8.33)	0.74

## Discussion

Prior to the 2013 WHO revision, MFS was classified as a myxoid subtype of malignant fibrous histiocytoma (MFH) ^1^. After this reclassification, MFS was recognized and treated as an independent tumor category. The management of MFSs presents several important challenges. First, MFS is characterized by a high propensity for recurrence, often necessitating limb amputation, which underscores the complexity of its treatment. Second, the absence of histologic findings that could be instrumental in predicting local recurrence further complicates the therapeutic approach. Additionally, the therapeutic options available for patients with MFS, particularly after multiple recurrences, are limited and often unsatisfactory. In this study, we performed a comprehensive analysis of LR outcomes and associated factors within a specific cohort of 52 patients diagnosed with MFS. This investigation was designed to contribute valuable insights into the understanding and management of this complex and often recalcitrant malignancy.

The median follow-up time for patients diagnosed with MFS was 52 months, with a corresponding five-year survival rate of 75%. This five-year OS rate aligns with the previously reported range of 61% to 77%^3–5^. The time to LR was observed to vary between 18 and 27 months, although instances of LR have been documented up to 8 years following initial treatment. The LR rate within the studied population was 48%, and multiple recurrences have been recorded in a substantial number of patients.

Multivariate analysis revealed that a variety of patient and clinical characteristics influence the likelihood of mortality (LR) in individuals with MFS. Numerous studies to date have identified factors such as surgical margins and location at diagnosis as significant predictors of LR^15^. These associations were corroborated in our research. In contrast to the findings of previous studies, which described superficial tumors as having no substantial impact on recurrence^16^, our study revealed that superficial tumors were associated with a heightened probability of recurrence.

Surgery represents the primary therapeutic intervention for MFS, and the importance of clear surgical margins has been extensively delineated in the literature ^17,18^. In this study, margins were precisely delineated during pathological assessment by a dedicated pathologist. The surgical specimen was examined by the operating surgeon. Margins were inked and individually sampled, and the closest margin was microscopically categorized as positive (presence of tumor within 1 mm from the inked surface) or negative (absence of tumor within 1 mm from the inked surface)^19,48^. Our study reinforces the critical role of negative margins in determining LR outcomes. Often, however, achieving sufficient cutting margins proves challenging, particularly when proximate to vital structures. Some scholars have advocated for the use of postoperative radiotherapy^20,21^ in patients with insufficient incisal margins, as a means to increase the likelihood of controlling recurrence. However, the practical role of radiotherapy in treating MFS remains unclear. In this study, due to the high degree of heterogeneity and small number of patients, our univariate analysis of radiotherapy and recurrence did not yield statistically significant results. However, the overall recurrence rate among patients who underwent postoperative radiotherapy was 30%, a figure that is notably lower than the aggregate recurrence rate. It must be noted, however, that this study did not delve into the specific impact of radiotherapy on patients with positive incisal margins, owing to the limited number of cases and the scarcity of patients with positive incisal margins. The infrequency of MFS renders prospective studies challenging to conduct, necessitating multicenter collaborative investigations to further elucidate the role of radiotherapy in future research. Importantly, none of the patients who underwent amputation experienced LR, underscoring that complete eradication of the disease is paramount for effective local control.

Tumor location is a notable factor that has been recognized to influence the likelihood of local recurrence. In our study, univariate analysis revealed a statistically significant difference in the probability of recurrence between patients with tumors located in the trunk and those with tumors located in the extremities (*P* < 0.05), corroborating previous findings^16^. However, in our multivariate analysis, this factor did not exhibit statistical significance. We hypothesize that the tumor site may be intricately linked to the resection margin and could contribute to tumor recurrence via its influence on the resection margin. The ability to achieve complete tumor resection may be compromised when dealing with tumors situated in the trunk due to their proximity to vital structures or inadequate soft tissue coverage. Such constraints may lead to the presence of residual tumor tissue, consequently elevating the risk of recurrence. Moreover, the anatomical limitations of the trunk region preclude the option of amputation as a means to ensure adequate margins. Consequently, patients with tumors located in the trunk require a comprehensive preoperative evaluation, coupled with multidisciplinary consultation. This integrated approach is designed to facilitate maximal tumor resection and may be complemented by adjunctive interventions such as postoperative radiotherapy.

Myxofibrosarcomas are categorized into two distinct subtypes: superficial and deep^22,23^. Superficial lesions are defined as those situated within the dermal or subcutaneous layer, while deep lesions are characterized as either intramuscular or subfascial. Superficial lesions are prone to infiltration, in contrast to deep lesions, which typically manifest as a singular, discrete masses with a nodular appearance, that extends longitudinally^24^. A preponderance of studies has demonstrated that myxofibrosarcomas exhibit a greater incidence of superficial lesions than other soft tissue sarcomas^23,25^, a finding corroborated by our data. Consequently, in this study, we stratified the subjects into two groups based on the depth of the mass to investigate the correlation between tumor depth and recurrence. Through univariate analysis, we ascertained that superficial tumors exhibited a greater propensity for recurrence than deep tumors. Subsequently, we integrated tumor depth into a multivariate logistic regression model, and deduced that superficial tumor depth was an independent predictor of tumor recurrence. The prevailing consensus attributes this phenomenon to the absence of a pseudocapsule in the superficial subtype, thereby enabling myxofibrosarcomas to extend along fascial planes and invade adjacent tissues, this phenomenon is also referred to as the “tail sign”^26^, resulting in inadequate excision. Moreover, superficial tumors are easy to treat at nonprofessional tumor centers, further improving the possibility of intratumor resection. This mechanism partially explains the elevated rate of locally recurrent disease within the unplanned excised subgroup, as this cohort was more likely to have a higher intralesional excision rate and a greater proportion of superficial tumors. Conversely, the deeper and more discrete the tumors, the more readily identifiable they become, consequently, they are less likely to be operated upon by a nonsarcoma specialist.

A planned operation was defined as a procedure that was thoroughly evaluated at our center, planned, and executed according to the results of comprehensive imaging examinations. In contrast, an unplanned operation refers to a procedure performed at another hospital without detailed evaluation, followed by referral to our facility for further intervention. In this study, we found that the likelihood of recurrence in patients who underwent planned surgery was significantly lower than that in patients who underwent unplanned surgery. Myxofibrosarcomas are often misdiagnosed due to their relative rarity and asymptomatic superficial presentation. This can lead to initial treatment at nonspecialized facilities, frequently resulting in unplanned marginal or intralesional resection. Such primary inadequate resections may facilitate the dissemination of tumor cells via hemorrhage and edema beyond the primary tumor site, culminating in elevated rates of LR. Consequently, some scholars have advocated immediate surgical re-resections to attain superior oncological outcomes, although this strategy has been contested due to the potential for increased patient morbidity^27,28^. Studies revealed that 92.6% of patients who underwent unplanned surgery initially still harbored viable tumors within the scars, reinforcing our stance that scars and contaminated tissue necessitate further resection. This consideration is particularly salient, as residual tumors in specimens designated for resection have been correlated with a heightened risk of LR^29^. Nevertheless, even subsequent to extended resection, the recurrence rate among patients who undergo unplanned surgery remains suboptimal. Research indicates that standardized adjuvant radiotherapy may ameliorate the recurrence rate in such patients, albeit not to the level achieved with specialized treatment at a sarcoma center from the outset. Several studies have corroborated the association between unplanned surgery and adverse prognosis and recurrence. For instance, Vos et al. demonstrated that surgery for low-grade and deep-seated STS in a high-volume center (≥ 20 resections annually) enhanced survival compared to procedures performed in a low-volume hospital (1–9 resections)^30^. Kikuta et al. reported similar findings in a cohort of 100 MFS patients, and identified primary unplanned resection at nonspecialized facilities as a risk factor correlated with poor prognosis^17^. Furthermore, Blay et al. established a positive correlation between surgery on STS and visceral sarcomas in a sarcoma expertise center and both local relapse-free survival and overall relapse-free survival^31^. Therefore, timely referral to a specialized sarcoma center may constitute an efficacious strategy for enhancing the survival prospects of patients with sarcoma.

We conducted a thorough analysis of laboratory findings on the initial postoperative day for all participants in our study. Given the necessity of extensive tumor resection, most patients exhibit varying degrees of postoperative creatine kinase elevation compared to their preoperative creatine kinase levels. However, it's crucial to note that preoperative creatine kinase levels vary among individuals. To mitigate the impact of individual differences, we focused on the disparity between postoperative and preoperative creatine kinase levels. Remarkably, we observed a lesser magnitude of creatine kinase elevation in recurrent patients than in their nonrecurrent counterparts. Consequently, we performed an ROC analysis of the difference in creatine kinase levels before and after surgery for all 52 patients, determining a cutoff value of 148 U/L for CK levels. We operationalized values below the cutoff as "low CK levels" and values surpassing the cutoff as "high CK levels”. This approach allows for a nuanced understanding of the creatine kinase dynamics in our cohort, enhancing the precision and clinical relevance of our findings. Our analysis revealed that serum CK levels were notably lower in patients who subsequently experienced relapses than in those who did not. Furthermore, subsequent multivariate analysis established low CK levels as an independent prognostic factor for MFS recurrence. The CK system plays a key role in cellular energy buffering and transport^32^. In vertebrates, CK has four isoforms expressed in a tissue-specific manner, and in this study, all patients were excluded from having myocardial or brain diseases. Recent literature has shown a progressive decrease in phosphocreatine, creatine, and CK levels during the transformation of skeletal muscle to sarcoma^33^. In the inflammatory milieu induced by tumors, TNF-α-triggered NF-κB activation suppresses the post-transcriptional expression of the myogenic differentiation Factor D (MyoD) mRNA. A regulatory site for MyoD in the CK-MM promoter has been identified, and the inhibition of MyoD synthesis may result in diminished CK-MM activity in muscle tissue^34,35^. Moreover, CK levels exhibit a noteworthy increase during tumor regression^36^. This explains the observed low serum creatine kinase levels in patients with MFS, suggesting that a less pronounced increase in creatine kinase in recurrent patients may indicate the presence of residual tumors. Moreover, CK is associated with immune status^37^. Ectopic CK expression increases ATP levels and enhances the phosphorylation of TCR signaling proteins. CK intensifies tyrosine phosphorylation of Lck and Zap70 and elevates phosphorylation levels of p38, JNK, and Erk1/2 in the presence of TCR stimulation. CK not only enhances T-cell activation, proliferation, and cytokine secretion but also plays a crucial role in regulating thymocyte development by modulating TCR signaling strength^38^. Consequently, low CK levels may signify a compromised state of immune responsiveness. The residual tumor cells, compromised immune system, and depletion resulting from surgery collectively create a conducive environment for tumor recurrence. Additional data are essential for validating these findings, and further fundamental research is needed to determine the underlying mechanisms involved.

Concurrently, we observed elevated serum lactate dehydrogenase (LDH) levels in the majority of patients with myxofibrosarcoma. LDH plays a crucial role in the Warburg effect, a phenomenon prevalent in cancer cell metabolism. A literature review revealed that LDH levels were associated with the prognosis of specific sarcomas. Elevated LDH levels have been identified as a significant predictive factor for disease-free survival (DFS) or overall survival in patients with osteosarcoma^39^ and Ewing sarcoma^40^. Furthermore, LDH levels serve as diagnostic, prognostic, and predictive markers for therapeutic response in various cancers^41^, including renal cell carcinoma, nasopharyngeal carcinoma, melanoma, prostate cancer, colorectal cancer, and lung cancer^42^. However, due to the occurrence of metastasis in only four patients and the eventual death of four individuals in this cohort, establishing a definitive relationship between LDH and prognosis was challenging. Consequently, our analysis focused solely on the association between LDH and recurrence. We defined the cutoff value for serum lactate dehydrogenase as 253 IU/L, categorizing values below this threshold as low LDH and those above as high LDH. Despite the use of both univariate and multivariate analyses, statistically significant results were not obtained. This finding suggested that the relationship between LDH and MFS recurrence may not be statistically significant. Nevertheless, considering that MFS is a malignant tumor with a high recurrence rate and relatively low mortality, further exploration of the relationship between LDH and prognosis has meaningful implications.

In our study, while males exhibited a slightly greater incidence of MFS than females did, sex differences were not identified as a significant prognostic factor. Interestingly, we observed that males had a significantly greater LR rate than females with MFS. This observation contrasts with most recent studies; for instance, Kaya et al. noted a nonsignificant increase in local recurrence-free survival in female patients^43^. One potential explanation for this discrepancy could be that male cancer patients generally have shorter survival durations than their female counterparts. However, this difference in survival tends to diminish in elderly populations^44^. Biologically, there is currently no established explanation for the observed difference in LR rates among MFS patients based on sex. While sex is a nonmodifiable factor, recognizing its potential impact on survival can be instrumental in formulating accurate prognostic assessments and devising optimal treatment plans.

Larger tumors are predisposed to a higher recurrence rate, as the complexity and extent of surgery can potentially lead to inadequate resection. Several scholars have suggested that larger tumor size is also a negative predictor of distant metastasis^45^. Simultaneously, high-grade tumors exhibit a greater propensity for recurrence. However, in our study, neither the tumor diameter or the tumor grade significantly affected the prediction of recurrence. Several factors may contribute to this lack of significance. First, the limited number of patients and the presence of numerous confounding variables may have impeded the attainment of statistically significant results. Second, patients with smaller tumor volumes and radiographically lower grades may be more inclined to undergo unplanned surgical interventions at external medical facilities. Inadequate excision in such cases could result in the dissemination of tumor cells beyond the primary site, facilitated by bleeding and edema, consequently leading to a higher recurrence rate and a less favorable prognosis. This may even equate the recurrence rate to that observed in larger, high-grade tumors, thereby obscuring significant statistical differences. The majority of retrospective studies on myxofibrosarcoma have not demonstrated benefits from chemotherapy or have been limited by small sample sizes^5,46,47^. Pogkas et al. also affirmed that chemotherapy did not enhance overall survival (OS), local recurrence-free survival (LRFS), or distant metastasis-free survival (DMFS)^45^. No significant improvement was observed in patients with tumors ≥ 10 cm. In our study, only three patients received chemotherapy, primarily consisting of anthracycline-based drugs and ifosfamide, supplemented with targeted drugs based on next-generation sequencing (NGS) results. The median progression-free survival (PFS) was only 2 months, and we lack sufficient data to validate the effectiveness of chemotherapy. Expanding the sample size is imperative for future investigations.

To further enhance the understanding of LR outcomes in myxofibrosarcomas and potentially improve them, several future possibilities not examined in this study warrant consideration. These include in-depth insights into the molecular drivers of MFS, an exploration of the immune microenvironment, and the application of advanced imaging techniques, possibly even during primary surgery, to augment R0 resection rates. Future research endeavors, such as case–control studies or prospective cohort studies, are recommended to elucidate the prognostic and predictive factors that determine the local recurrence rate in patients with MFS. Such studies should be designed to collect more comprehensive information, encompassing aspects such as social and environmental exposures, radiomics, and genomics. By integrating these diverse factors, a more holistic understanding of MFS recurrence may be achieved, paving the way for more targeted and effective interventions.

This study is subject to several limitations that warrant acknowledgment. First, the retrospective design inherently introduces known constraints. Given that this was a non-randomized analysis of prospectively collected data, intrinsic selection biases concerning patient allocations may have influenced the results. However, it is worth noting that patient demographics and tumor characteristics were largely comparable, mitigating some of these concerns. Second, the study's small sample size constitutes a important limitation. This constraint is largely attributable to the relative rarity of this particular tumor subtype. Future meta-analyses will be essential to overcome the challenges posed by the inevitably low-powered studies of this STS subtype. These analyses will further validate and evaluate the optimal treatment strategies for myxofibrosarcomas. Finally, the limited follow-up time in this study restricts the availability of sufficient prognostic results. Consequently, the prognostic factors were not analyzed in depth, and an extension of the follow-up period will be necessary to supplement the data and provide a more comprehensive understanding of the long-term outcomes. In conclusion, while these limitations must be considered when interpreting the findings, this study still offers valuable insights into the characteristics and treatment of MFS, and lays the groundwork for future research in this area.

## Conclusion

Myxofibrosarcomas present a challenging management conundrum due to their unpredictable clinical course. Currently, ensuring tumor-free surgical margins is of paramount importance. Moreover, the prognosis of patients with sarcoma can be improved by transferring patients with superficial tumors in the trunk to professional sarcoma centers as soon as possible. However, the future of tumor management lies in developing a greater understanding of the molecular basis of this tumor to develop and direct therapy accordingly.

## Data Availability

The datasets generated during and/or analysed during the current study are available from the corresponding author on reasonable request.
